# Evaluating the substitutability of fruit versus menthol and tobacco-flavored e-cigarettes on combustible cigarette cessation: Randomized clinical trial protocol

**DOI:** 10.1016/j.conctc.2026.101652

**Published:** 2026-06-06

**Authors:** Olivia Klapec, Andrew A. Strasser, E. Paul Wileyto, Janet Audrain-McGovern

**Affiliations:** a Department of Psychiatry, Perelman School of Medicine, University of Pennsylvania, Philadelphia, PA, USA; b Department of Biostatistics and Epidemiology, Perelman School of Medicine, University of Pennsylvania, Philadelphia, PA, USA

**Keywords:** e-cigarettes, Flavoring, Combustible cigarette smoking, Smoking cessation

## Abstract

While regulations have restricted the sale of pod-style fruit-flavored e-cigarettes, observational and clinical data suggest that their use is associated with smoking cessation among adults. Surprisingly, no randomized clinical trials have tested whether fruit flavors affect smoking outcomes compared with those authorized for sale, and, if so, why. Adults who smoke cigarettes daily, who have had multiple prior unsuccessful quit attempts, and who are interested in quitting smoking by switching to an e-cigarette will be recruited. Eligible adults will be randomized to fruit, menthol, or tobacco-flavored e-cigarettes. The baseline smoking rate will be established on days 1-5. Laboratory visits on days 6 and 7 will assess flavor-associated subjective reward and the reinforcing value of flavored e-cigarettes relative to combustible cigarettes. Participants will then receive an e-cigarette device and a 6-week supply of nicotine pods (5% nicotine) in their assigned flavor, and a structured action plan to quit smoking by switching to the e-cigarette. The primary outcome is the longitudinal daily cigarette count from baseline through the end of the six-week switch period (primary endpoint) and at the 26-week follow-up (secondary endpoint). Complete switching will be verified with a carbon monoxide value < 5 ppm. Secondary outcomes include subjective reward, relative reinforcing value, and positive affect. This trial will determine if fruit-flavored e-cigarettes facilitate initial and sustained switching more than menthol or tobacco, whether greater rewarding and positively reinforcing effects drive switching, and if the unavailability of fruit-flavoring may foster continued smoking for those unable to quit with FDA-approved medication.

This trial was registered at CT.gov (NCT06264154) https://clinicaltrials.gov/study/NCT06264154 on February 16, 2024.

## 1 Introduction

Nearly 70% of adults who smoke cigarettes (AWS) want to quit smoking [1], and 55% report trying to quit at least once in the past year, yet over 90% fail [2], despite using smoking cessation medication [1]. Indeed, the average smoker will attempt to quit smoking at least 30 times before abstaining for 12 months or longer [3,4]. These attempts typically occur over decades of smoking, carcinogen and toxicant exposure, resulting in 480,000 deaths annually [5,6].

As highlighted in the Surgeon General's Report [5], helping AWS who cannot quit smoking switch to less harmful noncombustible nicotine-containing products, such as e-cigarettes [[7], [8], [9], [10], [11], [12], [13], [14], [15], [16]], has the potential to reduce this health burden dramatically [5,7,16]. Substituting e-cigarettes for combustible cigarettes might only be possible for AWS persistently if e-cigarettes are accessible and appealing [7]. The Food and Drug Administration banned the sale of flavored pod-style e-cigarettes, other than tobacco and menthol, given their appeal to youth [17]. Harm reduction proponents have advocated for the continued availability of e-cigarette flavors to appeal to and aid AWS who have been unable to quit with traditional methods [7,18,19]. Yet there are no prospective studies of the effect of flavor on initial and sustained switching from combustible to electronic cigarettes.

Converging lines of research provide a strong scientific premise for a prospective investigation of the role of fruit flavor in the substitutability of e-cigarettes for combustible cigarettes among AWS persistently. Survey studies have observed greater fruit-flavored e-cigarette use among AWS who fully substituted e-cigarettes for combustible cigarettes [20]. Clinical trials of tobacco/menthol-flavored e-cigarettes for smoking cessation have shown modest or short-lasting effects [21,22], while a trial where AWS predominantly self-selected fruit flavors showed long-lasting effects of e-cigarettes versus nicotine replacement [18].

According to Behavioral Economic Theory, e-cigarettes will substitute for combustible cigarettes if they have comparable reinforcer efficacy [23,24], meaning they must be subjectively rewarding, relatively reinforcing, and capable of increasing positive affect [7]. Research suggests that through these mechanisms, fruit-flavored e-cigarettes may enhance the substitutability of e-cigarettes for combustible cigarettes among AWS. First, the sweetness of fruit flavors has the largest effect on subjective reward [25], and subjective reward increases as the concentration of fruit flavor increases [26]. Second, fruit-flavored e-cigarettes are vaped longer, more deeply, and more frequently than other e-cigarette flavorings [23,27,28]. Third, fruit-flavored e-cigarettes enhance nicotine's rewarding and reinforcing effects in smokers [23,29] compared to non-fruit-flavored e-cigarettes. Finally, e-cigarettes with fruit flavoring, especially one's brand, increase positive affect [27], and use maintains positive affect among abstinent AWS [30,31].

## 2 Aims and hypotheses

The current study (NCT06264154) has two aims. Aim 1 seeks to evaluate the effects of fruit-flavored versus tobacco-flavored and menthol-flavored e-cigarettes on combustible cigarette smoking among adults who have persistently smoked. We hypothesize that participants randomized to fruit-flavored e-cigarettes will be more likely to switch from combustible cigarettes to e-cigarettes during the 6-week switch phase and at the 26-week follow-up than participants randomized to tobacco-flavored or menthol-flavored e-cigarettes. Complete switching, partial switching, and no switching will be measured. Aim 2 seeks to examine whether the rewarding and reinforcing effects of e-cigarette flavoring mediate switching behavior. We hypothesize that fruit-flavored e-cigarettes will produce greater subjective reward, relative reinforcing value, and positive affect than tobacco-flavored or menthol-flavored e-cigarettes. In turn, greater subjective reward, relative reinforcing value, and positive affect will predict fewer cigarettes smoked per day during the 6-week switch phase and at the 26-week follow-up.

## 3 Methods

### 3.1 Overview of study design

**Fig. 1:**
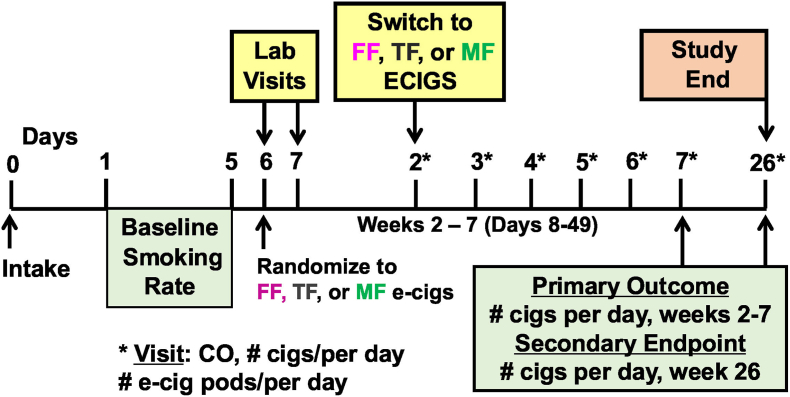
Overview of study design.

The proposed study will evaluate whether fruit-flavored (FF: watermelon and blueberry) versus traditional-flavored e-cigarettes [tobacco (TF) or menthol (MF)] more readily substitute for combustible cigarettes and if the rewarding and reinforcing effects of flavoring facilitate changes in smoking behavior among 210 adults who have persistently smoked cigarettes. Eligible adults will be randomly assigned 1:1:1 to FF (n = 70), TF (n = 70), or MF (n = 70) and participate in 9 visits across 26 weeks (see Fig. 1). Baseline cigarette smoking rate will be established over days 1-5. After overnight smoking abstinence, a laboratory visit on day 6 will assess e-cigarette flavor-associated subjective reward, affect, craving relief, and withdrawal relief. After overnight smoking abstinence, a laboratory visit on day 7 will assess the reinforcing value of the assigned e-cigarette flavor relative to combustible cigarettes.

Participants will then receive instructions and an action plan to switch to e-cigarettes for the following 6 weeks (weeks 2-7). Participants will collect their used cigarette filters and their used e-cigarette pods daily to assess consumption of cigarettes per day (CPD) and pods per day (PPD). The primary outcome will be the longitudinal daily count of cigarettes from baseline to the end of the 6-week e-cigarette switch phase. A secondary endpoint will be cigarettes per day from week 8 to week 26, measured by timeline-follow-back (TLFB). Complete switching will be verified with a carbon monoxide (CO) value < 5 ppm. Secondary outcomes include subjective reward, relative reinforcing value, and positive affect. The protocol was approved by the University of Pennsylvania's Institutional Review Board (854051) and participants provided written informed consent.

### 3.2 Participant eligibility

Study participants (≥21 years old) will be 210 male and female adults who have persistently smoked cigarettes and are motivated to switch from combustible cigarettes to e-cigarettes. Persistent smokers are defined as those who have repeatedly tried to quit and failed to quit, even with the use of smoking cessation medication. To be eligible, potential participants must: (1) be English speaking; (2) report having smoked at least 5 cigarettes per day for the past 6 months and ever use of an e-cigarette; (3) have a CO ≥ 10 ppm; (4) be willing to switch to e-cigarettes for 6 weeks and use the assigned flavors.

Exclusion from study participation will be warranted due to: (1) regular use of any nicotine-containing products other than cigarettes; (2) regular e-cigarette use defined as ≥ 5 days/30 days; (3) current or impending (during study period) enrollment in a smoking cessation program; (4) current use of smoking cessation medication; (5) allergies to either propylene glycol or flavor additives; (6) current use of recreational drugs (other than nicotine and cannabis), current treatment for substance use, and alcohol use exceeding 20 standard drinks per week; (7) current or planned pregnancy or lactation; (8) history or current diagnosis of psychosis; and (9) serious or unstable disease in the past year (e.g., cancer, cardiovascular event).

### 3.3 Participant recruitment and screening (Day 0)

AWS will be recruited from the community through social media advertisements. Those who respond to the ads will undergo phone prescreening to determine whether they meet the inclusion and exclusion criteria. Interested and initially eligible adults will attend an Intake Visit to confirm final eligibility. After providing informed consent, eligibility will be verified by a negative pregnancy test for females of childbearing potential, a negative urine drug screen, and a CO level >10 ppm. Those who qualify will complete baseline assessments, including demographics, smoking history, e-cigarette use history, nicotine dependence, and depression and anxiety symptoms.

### 3.4 Baseline smoking assessment (Week 1, days 1-5)

Participants will be instructed to smoke as usual on days 1-5. They will receive five date-stamped zipped baggies [32,33]. A written instruction card for collecting and storing spent cigarette filters will be reviewed with each participant. Participants will collect all spent filters through day five and receive a monetary incentive ($35) to return the baggies [32,33]. Participants will return used cigarette filters in the date-stamped zipped baggies at the day 6 laboratory visit. Participants will be randomized to fruit, tobacco, or menthol-flavored e-cigarettes in an unblinded manner, with the computerized allocation sequence generated by the biostatistician and accessible only to the data manager. Randomization will be stratified by sex, nicotine dependence, depression/anxiety, and menthol cigarette status.

### 3.5 Laboratory assessments

#### 3.5.1 Flavor-associated subjective reward and positive affect (Day 6)

Participants will attend a laboratory session after overnight smoking abstinence (10 h; CO verified <10) on day 6 [23,34]. Cigarette craving, withdrawal symptoms, and positive and negative affect will be measured before e-cigarette exposure. They will receive instructions on how to use the e-cigarette before two e-cigarette exposures based on their flavor group assignment. On day 6, the traditional flavor groups will receive either two exposures of tobacco or two exposures of menthol flavoring, depending on their assignment. The fruit flavor group will receive blueberry and watermelon in a counterbalanced order. All exposures will have the same characteristics, only differing by flavor. Participants will take 14 puffs for the first exposure and 14 puffs for the second exposure. The exposures will be separated by 45 min [23,35]. After each exposure, participants will complete measures of subjective reward, craving, withdrawal, and affect. We chose to offer both watermelon and blueberry to participants randomized to fruit-flavored e-cigarettes to promote adherence if either flavor was unappealing. After this 1.25-h visit, participants will be compensated $40.

#### *3.5.2* *R*elative reinforcing value of e-cigarette flavoring *(Day 7)*

Participants will arrive at their visit at 9:00 a.m. after abstaining from smoking overnight (10 h; CO-verified <10 ppm). Participants will receive an introduction to a validated behavioral choice paradigm that assesses motivation to use an e-cigarette in their assigned flavor category (fruit, tobacco, or menthol) relative to combustible cigarettes [23]. For those randomized to fruit flavoring, the aim is not to quantify the reinforcing value of the fruit flavors. The fruit flavor with the highest rewarding value (determined on Day 6) will serve as the fruit flavor for the relative reinforcing value of flavoring (RRVF) assessment.

The RRVF will be assessed by asking participants to “work” by moving a computer mouse to hit targets on one of two computer screens to earn points toward either e-cigarette puffs or cigarette puffs. Using a concurrent schedule [34,36], participants will be told that they can switch between the two screens as often as they wish. Participants are instructed to move the computer mouse so that the cursor hits the targets (e.g., either an electronic or combustible cigarette image) [23]. Consistent with relative reinforcement paradigms, the reinforcement schedule for the e-cigarette earning screen will remain constant at a fixed ratio FR-25 (25 targets achieved to earn a point). In contrast, the reinforcement schedule for the cigarette-earning screen will increase (require more effort) with a progressive ratio schedule of PR-25x over 10 trials, such that 25, 50, 75, 100, 125, 150, 175, 200, 225, and 250 targets will have to be achieved to earn a point [34,36,37].

The computer task will continue until a participant completes 10 trials and earns a total of 10 points, from which they will earn either one puff of an e-cigarette for each point (i.e., up to 10 puffs of an e-cigarette) or one puff of a cigarette for each point (i.e., up to 10 puffs of a cigarette). E-cigarette and/or cigarette puffs will be taken at the end of the procedure to prevent satiation from influencing responding in subsequent trials. RRVF is defined in relation to responding for cigarette puffs. The breakpoint is the highest trial that a participant is willing to work to earn a combustible cigarette puff and reflects the reinforcing value of cigarette smoking relative to e-cigarette vaping of the assigned flavor [23,37]. To ensure that responding is based on reinforcer preference rather than departure from the lab, the choice task will be followed by a 1-h wait in the laboratory. After completing this 2-h visit, participants will be compensated $45.

### 3.6 Switching intervention

#### 3.6.1 E-cigarettes

We propose to use the NIDA Standardized Research Electronic Cigarette (SREC) for Clinical Research, manufactured by NJOY, LLC. The latest version of the SREC is a USB rechargeable closed device with a 400mAh battery permitting ∼200 (3-s) puffs per charge. The user cannot adjust the voltage or temperature settings. This pod-based system uses sealed pods pre-filled with 1.9 mL of e-liquid containing 5% nicotine by weight. Each pod provides ∼300 puffs. The e-liquid has a 0.77 PG/VG ratio and comes in four flavors: rich tobacco, menthol, blueberry, and watermelon. The pharmacokinetic profile of SREC is comparable to other pod-style e-cigarettes on the market (e.g., JUUL), which have been shown to deliver nicotine efficiently, similar to combustible cigarettes [[38], [39], [40]]. The SREC produces a consistent and characterized aerosol and was designed to serve as the standard for NIH clinical studies with human subjects.

#### 3.6.2 Action plan

Participants will receive a six-week supply of e-cigarettes, distributed weekly, according to their flavor assignment. Before beginning the switch phase, participants will meet with a research staff member to receive instructions on how and when to use the e-cigarette and to develop an action plan for switching [8,14]. The 30-min action plan will include preparing for switch day, managing smoking triggers, and garnering support for behavior change. Studies have shown that brief preparation for switching from combustible to e-cigarettes is associated with higher smoking abstinence rates at 8 weeks and greater reductions in harmful chemical exposure than participants not receiving instructions [8,14], even among smokers uninterested in quitting smoking immediately [8]. As such, the action plan enables all participants to begin with the same level of preparation while maximizing external validity.

#### 3.6.3 Switch period (Weeks 2-7, days 8-49)

Participants will leave the laboratory assessment on day 7 with instructions for e-cigarette use, a device, a charger, and a 7-day supply of pods matching their group assignment. The 7-day supply (days 8–14) will be based on baseline cigarettes smoked per day (1 pod per pack smoked), with an extra pod in case a participant cannot attend a scheduled appointment [8,14]. Participants assigned to the fruit flavor can choose blueberry and watermelon. Those assigned to tobacco or menthol will receive their respective flavors. As in previous studies involving tobacco product switching [32,35,41], participants will be instructed to switch completely from combustible cigarettes to e-cigarettes (Day 8). Complete switching instructions lead to larger changes in smoking behavior than ad libitum use [8] or gradual reduction [42].

A written instruction card for collecting and storing used pods will be reviewed with each participant. Participants will receive seven date-stamped zipped baggies to collect used pods daily [32,33]. They will return to the Center on days 14, 21, 28, 35, 42, and 49 to have their CO measured, return their used pods, and receive their next seven-day supply of pods and zipped baggies (no pods or baggies on day 49). Participants will earn a $20 incentive at each visit. They will collect all used pods through day 49. Although the expectation is that participants will only use e-cigarettes, it's realistic to expect some will also smoke combustible cigarettes. We will ask participants to collect their spent cigarette filters if they smoke and place them in a date-stamped baggie. Participants will receive a $10/day incentive for collecting used pods and spent cigarette filters.

### 3.7 Follow-up (Weeks 8-26)

The switch phase will conclude at the end of week 7. Participants will then receive information on where and how to buy common e-cigarette brands, with attention to nicotine content [43]. Details on open and closed e-liquid systems, including disposables and potential purchase locations, will also be provided [44]. While non-disposable menthol and tobacco-flavored pod-style e-cigarettes are widely available, fruit-flavored pod-style e-cigarettes are less accessible. Up to 39% of brick-and-mortar stores continue to sell them, and online stores remain a possible source [45]. Disposable fruit-flavored pod-style e-cigarettes are easily found in vape shops throughout the city area, as are open e-cigarette systems that allow adding fruit-flavored e-liquid.

Participants will complete an in-person follow-up at 26 weeks. This follow-up will allow an assessment of cigarette smoking behavior, ongoing e-cigarette use [14], and the characteristics of e-cigarette use (such as device type, flavor, quantity of use, and nicotine content). This will enable us to observe short-term and long-term sustained switching, delayed switching, shifts in dual use, and the return to exclusive smoking [46] as participants navigate the current and evolving tobacco marketplace.

### 3.8 Assessments

Table 1 summarizes the measured variables and their measurement time points. Covariates will include demographics, smoking history, e-cigarette use history, marijuana use, nicotine dependence as measured by the Fagerstrom Test for Nicotine Dependence [47], the modified Penn State Dependence Scale [48], carbon monoxide measured with iCO quit (Bedfont Scientific), depression symptoms measured with the 20-item Center for Epidemiological Studies - Depression scale [[49], [50], [51]], and anxiety measured with the Generalized Anxiety Disorder-7 [52,53].

**Table 1:** Schedule of study measures.

Measures	Laboratory Visits				
	Baseline	Day 6	Day 7	Weeks 2-7	Week 26
**Baseline Covariates**					
Demographics	X				
Smoking History	X				
E-cigarette History	X				
Nicotine Dependence	X			X	X
Cigarettes per day (days 1-5)	X				
Depression & Anxiety	X				
CO	X	X	X	XXXXXX	X
**Predictor Variables**					
Craving Relief		X			
Withdrawal Relief		X			
Negative Affect		X			
ECIG pods per day				XXXXXX	X
**Mediating Variables**					
Subjective Reward		X			
Relative Reinforcing Value of Flavoring			X		
Positive Affect		X			
**Outcome Variable**					
Cigarettes per day				XXXXXX	X

Predictor variables will include craving, measured by the 10-item Brief Questionnaire of Smoking Urges [54,55]; withdrawal symptoms, assessed with the Minnesota Nicotine Withdrawal Scale [56,57]; and negative affect, evaluated using the Positive and Negative Affect Schedule [[58], [59], [60]]. Additionally, e-cigarette use will be recorded by counting the daily e-cigarette pods returned over each of the 42 days of the switch period (days 8–49) [32,35,61]. The total number of pods used during the switch phase will be determined by summing fully and partially used pods. Partially used pods will be weighed and converted into a fraction of a pod used [61].

Mediating variables include the subjective rewarding value of e-cigarette flavor, measured with the Satisfaction Subscale of the modified Cigarette Evaluation Questionnaire adapted for e-cigarette use [23,[62], [63], [64]]; positive affect measured with the Positive and Negative Affect Schedule [[58], [59], [60]], and the relative reinforcing value of flavor measured with a validated computer-based choice paradigm, which evaluates the preference for flavored e-cigarette puffs versus cigarette puffs [37,65].

The primary outcome is the longitudinal daily count of cigarettes from baseline to the end of the e-cigarette switch phase. Daily cigarette consumption will be determined by counting the daily spent cigarette filters returned for each of the 42 days (days 8 – 49) [32,35]. Cigarette smoking behavior at the 26-week follow-up will serve as a secondary endpoint. Average CPD at the 26-week follow-up will be measured via a valid and reliable TLFB procedure [66]. A CO value < 5 ppm will verify combustible cigarette abstinence (complete switching) at these time points.

### 3.9 Adverse event monitoring

Adverse events are monitored weekly during the switch phase using a self-report checklist. This checklist covers expected side effects of e-cigarette use and allows participants to list symptoms they are currently experiencing that are not on the list (unexpected). Adverse events are documented based on factors like severity, frequency, and type, and then reported to the PI, the Institutional Review Board, and the National Cancer Institute, in accordance with regulations.

### 3.10 Statistical considerations

#### 3.10.1 Sample size

For Aim 1, hypotheses will be tested with 80% power using 5% two-sided alpha Bonferroni corrected to 2.5% for two group comparisons. The primary outcome is the longitudinal daily cigarette count from baseline (days 1-5) to the end of the e-cigarette switch phase (day 49), with week 26 as a secondary endpoint. We based the power on data from prior switching studies. A sample of 210 (70 per group) will allow us to detect an overall standardized slope of 0.19. To account for a ∼15% attrition rate [67], we will enroll ∼ 240 participants to have 210 who complete the 26-week follow-up. We expect the initial and sustained switch for participants randomized to fruit-flavoring to be greater than for participants randomized to tobacco or menthol flavoring. A sample of 70 per group enables us to detect a difference of ∼3.73 cigarettes per day (CPD). We will also be able to detect a difference in slopes of 0.13 (CPD per day change).

For Aim 2, a sample of 70 per group will allow us to detect a between-group effect size of 0.5. For the relative reinforcing value of flavored e-cigarettes (RRVF), this means we can detect a difference in breakpoint of 2.0. Positive affect will be assessed as a difference score with a standard deviation of approximately 7.0, yielding 80% power to detect a difference of approximately 3.7. Subjective reward has a standard deviation of 1.3, which allows us to detect a 0.68 difference. In turn, the continuous measures of RRVF, positive affect, and subjective reward will be used to predict changes in mean cigarette counts from baseline to the end of the switch phase and from baseline to the 26-week follow-up. The sample of 210 allows us to detect a standardized slope of 0.2. The actual standard deviation for the cigarette consumption change score is 7.1. The detectable slopes are 0.37 for RRVF, 0.20 for positive affect, and 1.07 for subjective reward.

#### 3.10.2 Data analysis

Prior to analyses, the data will be screened for entry errors and outliers, the extent and type of missing data will be assessed, and the most appropriate method for dealing with the missing data will be selected. For Aim 1, longitudinal cigarette counts (normalized as percent of baseline) will be analyzed using mixed models (Gaussian family) in the primary analysis. The model will include predictors of time (continuous or discrete), study phase (baseline, switch period, or follow-up), flavor group, candidate covariates, and predictors (e.g., e-cigarette use). It will be tested using the z-scores corresponding to the study phase by flavor group interaction terms with fruit-flavored as the reference. For an intent-to-treat (ITT) analyses, ITT will be defined as those randomized to a flavor group at day 6.

For Aim 2, measures are summary scores and difference scores and will be addressed using multiple regression methods. We will first test whether participants randomized to fruit versus tobacco or menthol-flavored e-cigarettes report greater subjective reward, greater increases in positive affect, and have a higher relative reinforcing value of e-cigarette flavoring using the t-score associated with the group difference. We will then test whether these effects predict fewer cigarettes smoked per day during the 6-week switching phase and at the 26-week follow-up.

Next, we will evaluate whether subjective reward, positive affect, and relative reinforcing value for entry into a model as predictors of change in cigarettes smoked per day (mean switch phase or follow-up cigarettes minus mean baseline cigarettes), using the significance of the corresponding t-score while controlling for craving, withdrawal, and negative affect relief. The series of regressions will occur within a structural equation model, with the effects of flavor (predictor) on cigarettes per day (outcome) across the switch follow-up phases mediated by subjective reward, positive affect, and the relative reinforcing value of flavor. We will compute the proportion of mediation for the indirect effects and evaluate the mediated paths for significance using the delta method and bootstrapped standard errors [[68], [69], [70]].

#### 3.10.3 Missing data and dropout

We anticipate approximately 15% of the original 240 participants will drop out by week 26. The primary analysis of Aim 1 will be all available data, which may include some intermittent missingness, and some dropout. Because we cannot determine the cause of missingness, we will follow the primary analysis with a sensitivity analysis, looking for evidence of bias in our estimates revealed by the application of various data models. Those models will include (1) completers only, (2) last observation carried forward, (3) missing equals return to baseline smoking, and (4) multiple imputation using the MICE method.

## 4 Discussion

Despite the public health and regulatory significance, the field has little understanding of the effects of e-cigarette flavoring on the likelihood of switching from combustible cigarettes to e-cigarettes. Such information is critical, as the availability of e-cigarette flavoring in the marketplace has been the subject of vigorous debate [19,71]. Converging lines of research provide a strong scientific premise for a prospective investigation of the role of fruit flavor in the substitutability of e-cigarettes for combustible cigarettes among adults who have been unable to quit smoking with traditional approaches. This protocol describes the first randomized clinical trial to assess the effects of e-cigarette flavoring on switching from combustible cigarettes to e-cigarettes, and the likely mechanisms driving initial and sustained switching.

This clinical trial features several well-reasoned and innovative design choices. For example, the hedonic and motivational mechanisms that are key indicators of abuse liability, namely subjective rewarding value and relative reinforcing value, are likely the same mechanisms that explain a noncombustible nicotine delivery product's ability to fully substitute for combustible cigarettes. As the influx of alternative nicotine delivery products in the marketplace continues, these variables could serve as an early indicator of their value as a cessation aid. These indices will be examined in the laboratory using well-validated behavioral choice paradigms [23,34], and then mapped onto cigarette smoking behavior in a real-world setting. Thus, both laboratory and field methods are used to address public health and policy-relevant research questions regarding e-cigarette flavoring. Additionally, we will evaluate whether the rewarding and positively reinforcing effects of fruit versus traditional flavoring drive switching behavior, while accounting for negative reinforcing effects, such as reduced craving, withdrawal, and negative affect.

The study focuses on AWS who have tried repeatedly, but unsuccessfully, to quit smoking. While this may lessen generalizability, this population is most suited to a harm-reduction approach [7,16,19]. These AWS have not been successful with medication and their likelihood of quitting is less when the same medication is used on sequential attempts [72]. With only three medications approved by the FDA over the past five decades, these individuals have few options. Research indicates adults are turning to e-cigarettes more frequently than any other medicinal smoking cessation product [73].

If the hypotheses are supported, it would suggest that the unavailability of fruit-flavoring in e-cigarettes would unintentionally support continued combustible cigarette smoking among adults who have not been able to quit using traditional approaches. The findings may help to catalyze thinking surrounding the need for regulatory precision (e.g., moving all non-medicinal nicotine to adult retail outlets) rather than a complete flavor ban [19,71,74] to protect youth from using e-cigarettes while promoting use among smokers unable to quit smoking otherwise. If the hypotheses were not supported, it would suggest that fruit-flavored e-cigarettes do not confer an advantage over traditional flavors for smoking cessation and that their absence from the market does not negatively affect AWS who want to quit through e-cigarette use.

### 4.1 Potential challenges

This study may face challenges, such as the need to offer and adapt some of the nine visits from in-person to virtual formats to ease the participant burden. The study e-cigarette, SREC, may become unavailable, requiring us to order the study supply at one time point or transition to another e-cigarette.

### 4.2 Conclusions

The current study is expected to yield new knowledge on whether fruit-flavored versus traditional-flavored (menthol, tobacco) e-cigarettes enhance the likelihood of successful switching, why they do so, and whether the effects are sustained over time.

## Ethics approval and consent to participate

The protocol was approved by the Institutional Review Board at the University of Pennsylvania (#854051). All procedures are conducted in accordance with the Declaration of Helsinki. The privacy rights of human participants will be observed and written informed consent will be obtained for participation.

## Availability of data and materials

No date was used for the research described in the article.

## Use of generative AI

None.

## Funding

This research is supported by the 10.13039/100000054National Cancer Institute, R01CA287474 (PI Audrain-McGovern). The funding source was not involved in the study design, data collection, data analysis, data interpretation, or the preparation of this manuscript.

## CRediT authorship contribution statement

**Olivia Klapec:** Investigation, Supervision, Writing – original draft, Writing – review & editing. **Andrew A. Strasser:** Investigation, Methodology, Writing – original draft. **E. Paul Wileyto:** Formal analysis, Writing – original draft. **Janet Audrain-McGovern:** Conceptualization, Funding acquisition, Investigation, Methodology, Project administration, Supervision, Writing – original draft, Writing – review & editing.

## Declaration of competing interest

The authors declare no conflicts of interest.

## Data Availability

No data was used for the research described in the article.

## Abbreviations

FDA: Food and Drug Administration
AWS: Adults who smoke
CPD: Cigarettes per day
ECIGS: E-cigarettes
FF: Fruit-flavored
TF: Tobacco-flavored
MF: Menthol-flavored
SREC: NIDA Standardized Research Electronic Cigarette
NIH: National Institutes of Health
CO: carbon monoxide
RRVF: relative reinforcing value of flavoring RRVF
TLFB: Timeline follow back
